# Therapeutic Potential of Amino Acids in Inflammatory Bowel Disease

**DOI:** 10.3390/nu9090920

**Published:** 2017-08-23

**Authors:** Yulan Liu, Xiuying Wang, Chien-An Andy Hu

**Affiliations:** 1 Hubei Collaborative Innovation Center for Animal Nutrition and Feed Safety, Hubei Key Laboratory of Animal Nutrition and Feed Science, Wuhan Polytechnic University, Wuhan 430023, China; xiuyingdk@foxmail.com; 2 Department of Biochemistry and Molecular Biology, University of New Mexico School of Medicine, Albuquerque, NM 87131, USA

**Keywords:** inflammatory bowel disease, animal models, amino acids

## Abstract

Inflammatory bowel disease (IBD), which includes both ulcerative colitis and Crohn’s disease, is a chronic relapsing inflammation of the gastrointestinal tract, and is difficult to treat. The pathophysiology of IBD is multifactorial and not completely understood, but genetic components, dysregulated immune responses, oxidative stress, and inflammatory mediators are known to be involved. Animal models of IBD can be chemically induced, and are used to study etiology and to evaluate potential treatments of IBD. Currently available IBD treatments can decrease the duration of active disease but because of their adverse effects, the search for novel therapeutic strategies that can restore intestinal homeostasis continues. This review summarizes and discusses what is currently known of the effects of amino acids on the reduction of inflammation, oxidative stress, and cell death in the gut when IBD is present. Recent studies in animal models have identified dietary amino acids that improve IBD, but amino acid supplementation may not be adequate to replace conventional therapy. The animal models used in dietary amino acid research in IBD are described.

## 1. Introduction

Inflammatory bowel disease (IBD) is a chronic, idiopathic and relapsing inflammation of the gastrointestinal tract with characteristics of severe diarrhea, electrolyte loss, bleeding and abdominal pain, and causes significant morbidity [1,2]. IBD also increases the risk of developing colorectal cancer [3]. Crohn’s disease and ulcerative colitis, the two types of IBD, have distinct pathological and clinical characteristics: Crohn’s disease can involve any part of the gastrointestinal tract, but most commonly affects the terminal ileum and perianal regions [2,4]. Ulcerative colitis usually affects the large intestine and the rectum [2,4]. Transmural inflammation is characteristic of Crohn’s disease, but in ulcerative colitis, inflammation is typically limited to the mucosa and caused by white blood cell infiltration [1,4]. Because of its complex etiology and complicated symptoms, IBD is very difficult to treat.

Genetics, environmental and microbial factors, components of the innate and adaptive immune systems, oxidative stress, and inflammatory mediators, are all known to be risk factors of IBD [4,5], but little is known about how they interact with each other in the progression of IBD. Experimental chemical-induced, immune-mediated, spontaneous, genetically engineered, and transgenic mouse and pig models can mimic the progression of IBD [5]. They have been used to study the pathophysiology and etiology of human IBD and for drug discovery and development. 

Amino acids are required for intestinal growth and maintenance of mucosal integrity and barrier function. They are used by small intestinal mucosal cells as essential precursors for metabolically active proteins, glutathione (GSH), nitric oxide, polyamines, and other molecules [6]. They are also used as building blocks of macromolecule synthesis for intestinal mucosal wound healing, and energy substrates of enterocytes [7]. The gut bacteria can utilize the amino acid to synthesize proteins and varieties of metabolites [8]. Amino acid metabolism in gut microbiota plays an important role in nutrition and physiology of the host [8]. Some studies, primarily conducted in rodents and pigs, are of interest because they have provided evidence that certain amino acids, particularly glutamine and arginine, may influence the progress of IBD [7]. They may act to reduce inflammation, oxidative stress, and the levels of proinflammatory cytokines. The current use of animal models in IBD research and the potential for use of specific amino acids in IBD treatment are reviewed below.

## 2. Animal Models of IBD—The Chemical-Induced Models

The complexity of IBD may account for the successful development of several different types of animal models. The chemical-induced models, include dextran sulfate sodium (DSS), trinitrobenzene sulfonic acid (TNBS), or acetic acid-induced models, are widely used experimentally because they are reproducible and easy to work with. In this section, the use of these models to study the pathogenesis of IBD and to test novel amino acid therapies are reviewed (Table 1).

### 2.1. DSS

DSS is a sulfated polymer that alters tight junction proteins, leading to the disruption of the intestinal barrier, and is toxic to epithelial cells. Results obtained in DSS-induced animal IBD models have helped to understand the pathogenesis of IBD and to screen potential therapeutic agents. DSS-induced IBD is simple and reproducible and results in symptoms resembling those of ulcerative colitis. The development of acute, chronic, or relapsing symptoms of IBD is dose-dependent, and the size of the DSS is key not only in the induction of colitis but also the location. A comparative study found that colitis developed in animals treated with 5 kDa and 40 kDa DSS but not 500 kDa, and severe colitis developed in the lower colon following administration of 40 kDa, whereas mild colitis developed in the cecum and upper colon after 5 kDa [25]. Various DSS dosage and duration are used to induce IBD in some animal models (Table 1) [9,10,11,12,13,14,15,16]. Following DSS administration, animals usually develop colitis with weight loss and severe, bloody diarrhea [11,13,16,26,27]. DSS colitis is characterized by mucosal ulceration, leukocyte infiltration, intestinal crypt distortion and epithelial hyperplasia [11,13].

It is believed that DSS colitis results in epithelial cell injury and increases the permeability of the intestinal mucosa to large molecules. DSS colitis is accompanied with dysregulation of the gut microbiota [28,29], and is associated with stimulation of innate and adaptive lymphoid elements and secretion of proinflammatory cytokines and chemokines [30,31]. The percentages of CD3^+^ T lymphocytes in Peyer’s patches, natural killer (NK) and B lymphocytes in mesenteric lymph nodes, and NK CD8^−^ cells in intraepithelial lymphocytes are elevated in DSS-treated animals [32]. Expression of P-selectin glycoprotein ligand-1, leukocyte function-associated antigen-1, and C-C chemokine receptor type 9 by T helper and cytotoxic T cells also increase after DSS treatment [10]. Tissue cytokine and chemokine levels, including interleukin (IL)-1α, IL-1β, IL-6, IL-17, granulocyte colony-stimulating factor, granulocyte-macrophage colony-stimulating factor, eotaxin-1, monocyte chemoattractant protein 1, macrophage inflammatory protein (MIP)-1α, and MIP-1β also change following exposure to DSS [14]. Redox status is also disturbed as shown by decreased GSH and catalase (CAT) and increased reactive oxygen species (ROS), malondialdehyde (MDA), nitric oxide and myeloperoxidase (MPO) [26,33]. Activation of the nuclear factor-κB (NF-κB) pathway has been linked with the pathogenesis of DSS-induced colitis [11], and DSS induces cell death signaling by modulating B-cell lymphoma (Bcl)-2 and Bcl-2-associated X protein (Bax) apoptosis factors [34,35], and receptor-interacting protein 3, mixed lineage kinase domain-like protein, and caspase-8 necroptosis factors [35,36,37]. Intestinal inflammation resulting from increased cell death has been reported to occur in IBD [34,36,38].

### 2.2. TNBS

TNBS elicits dysregulation of the intestinal immune system, and induces both acute and chronic colonic inflammation and ulceration [39]. Animal models that present characteristics similar to those of human IBD, predominantly Crohn’s disease, have been developed by injection of TNBS in ethanol. The model advantages include simple induction, rapid establishment, and development of reproducible, long-lasting colon damage accompanied by inflammatory cell infiltration and ulcer formation [40]. In the TNBS model, ethanol facilitates disruption of the intestinal barrier and helps TNBS enter the mucosal layer to induce colitis [41,42]. Various dosage combinations of TNBS and ethanol have been reported to induce IBD in various animal models (Table 1) [9,17,18,19,20,21]. 

Clinical phenotypes in TNBS/ethanol-induced animal models include diarrhea, weight loss, high mortality, and severe inflammation [9,39,43]. Massive bowel edema, and disruption of epithelial cells by large ulcerations are frequent, severe macroscopic injuries [9]; severe and intense transmural inflammation and/or diffuse necrosis, inflammatory granulomas, and submucosal neutrophil infiltration are also characteristic of this model [39]. 

Oxidative, inflammatory, and apoptotic damage may be among the causes of TNBS-induced colitis [44,45], leading to increased generation of prostaglandin E_2_ (PGE_2_) [18], increased MDA [44], NF-κB [44], MPO [46], nitric oxide synthase (iNOS) [18], and caspase-3 [44], increases of IL-1α, IL-1β, IL-4, IL-6, tumor necrosis factor (TNF)-α [17,46], and cyclooxygenase (COX)-2 [18], and decreased GSH in colon tissue [44]. Increased CD3^+^, CD4^+^ and CD8^+^ T lymphocytes in peripheral blood have also been reported in TNBS-induced colitis animal models [43].

### 2.3. Acetic Acid

Acetic acid-induced colitis is a common experimental IBD model. Animals treated with acetic acid develop many pathological and histopathological characteristics in common with human ulcerative colitis. Rectal application of acetic acid damages the mucosal epithelium and produces ulcerative colitis [47]. The severity of the mucosal lesions that develop in acetic acid-induced colitis depends on the acetic acid concentration and the length of exposure. Injection of 25% acetic acid into the gastric lumen causes larger ulcerative lesions than injection of 5% acetic acid [48]. Different concentrations of acetic acid and different exposure times have been reported to induce IBD in rat model (Table 1) [22,23,24]. Transmural necrosis in all layers of the bowel wall, severe neutrophil infiltration of the intestinal tissue, goblet cell depletion, edema, and submucosal ulceration are common manifestations of this model [23,49,50,51]. Bloody diarrhea [52], reduction of the intestinal mucus [51], decreased nucleic acid (DNA and RNA) and total protein content [51], and increased colon weight [23,51] and vascular permeability [50] have also been observed. MPO activity and MDA levels are elevated, and contents of GSH, superoxide dismutase (SOD) and CAT are significantly reduced in acetic acid-induced colitis [53,54]. Serum nitrate [53] and lactate dehydrogenase [50], caspase-3 [54,55], proinflammatory mediators iNOS, COX-2, IL-1β, IL-6, and TNF-α [49,50,51,55] were all significantly increased in acetic acid-treated animals. Acetic acid-induced colitis is also associated with changes NF-κB, inhibitor of κB (IκB) and IκB kinase expression [49].

The chemically induced IBD models are relatively easy and rapid to develop and can be used with wild-type mice or other normal animals. Thus, these models have been most commonly used to identify potential therapeutic agents [56]. However, the acute models may provide only limited information about the pathogenesis of IBD in humans, as the chemical damage to the intestinal barrier results in self-limiting inflammatory response rather than chronic disease [56]. Hence, these models are more relevant in research about acute inflammation. In fact, the intestinal microbiota signature may be different in animals suffering from chronic colitis [57]. In addition, these models may only partially reflect the human situation, e.g., in terms of genetics, environmental factors, dysbiosis, inflammatory response or disturbed permeability in human IBD [56]. Hence, findings in animal models of IBD may not always be consistent with observations in human IBD.

## 3. Potential Roles of Amino Acids in IBD

**Figure 1:**
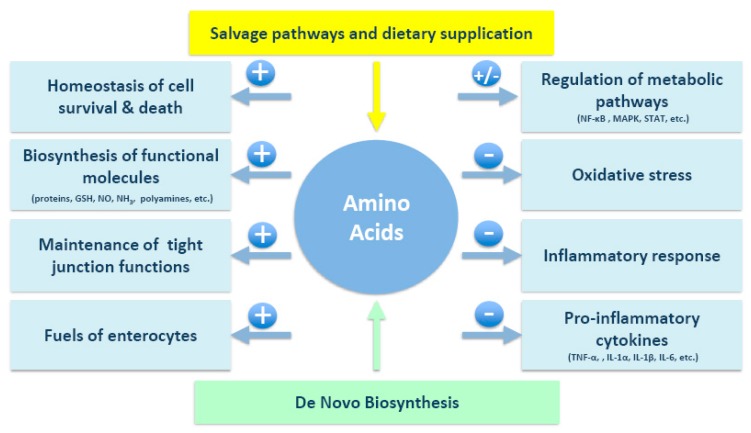
Projected mechanisms by which amino acids exert their beneficial effects on IBD. GSH, glutathione; IL, interleukin; MAPK, mitogen-activated protein; NF-κB, nuclear factor-κB; NO, nitric oxide; STAT, signal transducers and activators of transcription; TNF-α, tumor necrosis factor-α.

**Figure 2:**
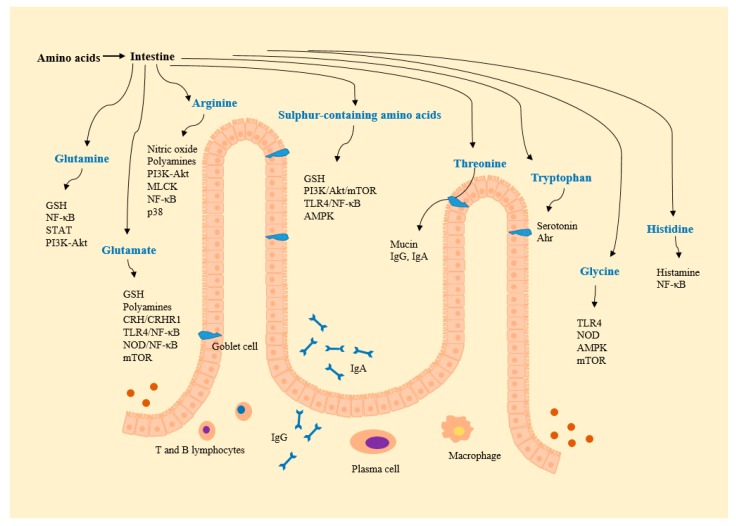
Amino acid-derived metabolites and amino acid-regulated signals. Ahr, aryl hydrocarbon receptor; Akt, protein kinase B; AMPK, AMP-activated protein kinase; CRH, corticotropin-releasing hormone; CRHR, CRH receptor; GSH, glutathione; MLCK, myosin light chain kinase; mTOR, mammalian target of rapamycin; NF-κB, nuclear factor-κB; NOD, nucleotide-binding oligomerization domain protein; PI3K, phosphoinositide-3-kinases; STAT, signal transducers and activators of transcription; TLR, toll like receptor.

Aminosalicylates, corticosteroids, and thiopurines can decrease the duration of active disease and contribute to the maintenance of IBD remission, but these treatments have adverse effects, especially with long-term use [58]. Novel adjunct therapies are thus needed to overcome the limitations of current pharmacological treatment. Interestingly, serum amino acid levels have been shown to differ in IBD patients and healthy controls, suggesting the existence of a link between amino acid profiles and IBD [12,14,59]. Moreover, malnutrition in IBD patients is common, and nutritional therapy is frequently used to overcome nutrient deficiencies and to alter the inflammatory status [58]. Amino acids, as therapeutic candidates, may ultimately help to maintain intestinal integrity in IBD patients (Table 2 and Figure 1 and Figure 2).

### 3.1. Glutamine

Glutamine is an important gut nutrient, required by enterocytes, and a precursor for biosynthesis of GSH, other amino acids, nucleic acids, and other biologically important molecules [72,73]. Glutamine supplementation is beneficial in improving disease symptoms and intestinal structure and barrier function [72,73]. This amino acid can increase intestinal-friendly microbiota (*Bacteroidetes* and *Actinobacteria*), while decrease pernicious bacteria (*Oscillospira* and *Treponema*) [74]. Its role in IBD is controversial [72,75,76,77,78], but the results of most glutamine studies support further evaluation as a potential therapeutic agent. In animal models, glutamine was found to protect against TNBS-, DSS- or acetic acid-induced intestinal damage and preserve gastrointestinal function. Weight loss [10], epithelium injury and loss [60], mucosal hypoplasia [60], submucosal and serosal fibrosis [17], colon edema [10], and disruption of colonic architecture [17] characteristic of chemically induced models were attenuated by glutamine administration. The evidence suggests glutamine may have antioxidant, antiapoptotic, and anti-inflammatory activity in IBD. In IBD models, glutamine has been found to decrease MDA level and caspase-3 activity and increase GSH level and heme oxygenase 1 expression [44,61]. The therapeutic effect of glutamine in experimental colitis results from a decrease in inflammation by modulation of NF-κB [49,62] and signal transducers and activators of transcription (STAT) signaling pathways [62], and from suppression of T cell migration [10,11]. Glutamine supplementation has been shown to regulate NF-κB and phosphoinositide-3-kinases (PI3K)-protein kinase B (Akt) signaling in vivo in a DSS-induced colitis model [12] and to enhance heat shock protein (HSP) expression both in vitro and in vivo in an experimental colitis models [15]. San-Miguel et al. [17] reported that glutamine supplementation prevented fibrosis development in TNBS-treated rats by down-regulating expression of collagen Iα2, collagen III, transforming growth factor-β, phosphorylated Smad3, platelet-derived growth factor, and connective tissue growth factor genes. Crespo et al. [45] reported that the protective effect of glutamine in colitis was associated with changes in the endoplasmic reticulum stress response (cytosine-cytosine-adenosine-adenosine-thymidine enhancer-binding protein homologous protein, binding immunoglobulin protein, and caspase-12), unfolded protein response signaling branches (activating transcription factor (ATF) 4, ATF6, and spliced X-box-binding protein-1), Jun N-terminal kinase, Bcl-2 family proteins, and caspase activation [45].

### 3.2. Glutamate

In most cells, the immediate enzyme-converted product of glutamine metabolism is glutamate [79], which is active as an excitatory neurotransmitter [80], participates in enterocyte oxidative metabolism [81], is a precursor of various polyamines, amino acids, and GSH [82], and regulates oxidative reactions [83] and metabolic pathways [83]. In an inflammation model, we previously demonstrated that glutamate could improve intestinal barrier function, alleviate inflammation and inhibit protein degradation via corticotropin-releasing hormone (CRH)/CRH receptor 1, toll-like receptor (TLR) 4 and nucleotide-binding oligomerization domain protein (NOD)/NF-κB, and mammalian target of rapamycin (mTOR) signaling, respectively [84,85]. Given its unique role in the gastrointestinal tract, glutamate may be an adjuvant IBD treatment with broad application, which has been confirmed by Li et al. [63]. In a rat model of ulcerative colitis, microinjection of glutamate into the hypothalamic paraventricular nucleus (PVN) promoted cell proliferation (increase of proliferating cell nuclear antigen-positive cells) and increased antioxidant levels (reduced MDA content and elevated SOD activity), inhibited apoptosis (upregulation of Bcl-2 protein and downregulation of Bax and caspase-3 protein), and the expression of proinflammatory factors TNF-α and IL-1β [63]. The glutamate mechanism of action may depend on binding to a glutamate receptor on the membrane of PVN neurons.

### 3.3. Arginine

Arginine is a conditionally essential amino acid and a precursor of numerous physiologically active molecules including nitric oxide and polyamines. Dietary arginine induces a shift in the Firmicutes:Bacteroidetes ratio to favor Bacteroidetes in gut [86]. Arginine has important functions in intestinal nutrition and health, is considered as a crucial nutrient for fetuses and neonates, and, especially in the disease states [87]. A study that assessed the expression of arginine transporters and downstream metabolic enzymes, revealed altered arginine metabolism in the gut tissue of IBD patients [88]. Tissue arginine level is also decreased in IBD patients [88], suggesting that dietary arginine supplementation might compensate the arginine deficiency in IBD tissues. Arginine administration has been shown to maintain normal intestinal physiology and to facilitate mucosal healing when the intestine is affected by inflammation [89,90,91]. Arginine treatment of mice with DSS colitis was shown to reduce mucosal permeability, the number of MPO-positive neutrophils, and expression of proinflammatory cytokines and chemokines and to increase iNOS activity [14]. Other evidence from animal models indicates that dietary arginine may improve clinical and biochemical parameters in DSS colitis via effects on PI3K-Akt and myosin light chain kinase signaling [12]. Histopathological evaluation has revealed alleviation of macroscopic and microscopic damage of colonic tissues induced in an acetic acid IBD model by arginine supplementation [64]. The protective effects were attributed to arginine’s nitric oxide donating property and modulation of NF-κB expression [64]. When it was combined with glutamine, arginine had synergistic effects on reduction of major proinflammatory cytokines mediated by the NF-κB and p38 pathways in active Crohn’s disease [92]. Oral coadministration of garlic and arginine attenuated the alterations induced by acetic acid, and increased GSH content [53].

### 3.4. Sulfur-Containing Amino Acids (Methionine and Cysteine)

Methionine is known to improve intestinal antioxidant capacity, villus morphology and development, transepithelial electrical resistance, and abundance of tight junction proteins, and to reduce plasma urea nitrogen [93,94]. Interestingly, reduction in dietary methionine intake has also resulted in improvement of epithelial barrier function [95] and suppression of colonic tumor development [96]. Consistent with this, rats fed with methionine-restricted diet were found to have higher transepithelial electrical resistance and claudin-3 protein expression, and decreased severity of epithelial injury in an ulcerative colitis model induced by DSS [97]. However, methionine-deficient diets can cause enterotoxigenic *Escherichia coli* adhesion, reduce autophagy and accelerate death of intestinal epithelial cells infected with enterotoxigenic *Escherichia coli* [98]. *S*-adenosylmethionine, a GSH precursor and the activated form of methionine, can inhibit lipopolysaccharide-induced TNF-α expression in macrophages [99]. Colon lesions, increased serum amyloid A and TNF-α, and cytoskeleton damage of cells in the intestinal mucosa in DSS-treated mice were reversed when treated with S-adenosylmethionine [27]. 

Cysteine is a sulfur-containing amino acid, and although high intake of cysteine is extremely toxic in animals [100], dietary cysteine has important anti-inflammatory and antioxidation activities [101]. Kim et al. [16] reported that cysteine attenuated IBD symptoms by restoring gut immune homeostasis and susceptibility of activated immune cells to apoptosis. Cysteine decreased chemokine expression, neutrophil influx, expression of TNF-α, IL-6, IL-12p40, and IL-1β inflammation-related cytokines and FLICE-like inhibitory protein and Bcl-xL prosurvival genes, and increased caspase-8 expression [16].

*N*-acetylcysteine (NAC), the precursor of cysteine, is rapidly metabolized by the gut to generate GSH [102], and improve intestinal bacteria with an increase in Lactobacillus and Bifidobacterium counts and a decrease in *Escherichia coli* count [103]. NAC is protective of intestinal health, and has beneficial effects in pathological conditions. Supplementation with exogenous GSH or NAC has been shown to attenuate dimethyl fumarate-induced necroptosis of CT26 murine colon adenocarcinoma cells [104]. In a pig model, dietary supplementation with 500 mg/kg NAC increased goblet cell number, and protein/DNA ratio, claudin-1 protein expression, and prevented increase of intraepithelial lymphocytes and caspase-3 protein expression in the colonic mucosa [54]. Cetinkaya et al. [65] demonstrated that intraperitoneal or intrarectal NAC decreased MPO activity and MDA level, elevated GSH level and SOD and CAT activities in an acetic acid-induced rat IBD model. Others have shown that NAC attenuated macroscopic colonic damage and histopathologic changes, decreased colonic MPO activity and ROS, upregulated paraoxonase 1 (an antioxidant enzyme), and scavenged oxygen-derived free radicals in a mouse DSS colitis model [24,26]. In addition to restoring normal levels of antioxidant enzymes, NAC administration also decreased TNF-α, IL-1β, IL-6 proinflammatory cytokines [24,54]. Rectal administration of 13 mg/kg NAC limited TNBS-induced intestinal damage in rats by suppressing COX2 gene expression and PGE_2_ level [18]. NAC (0.2%) has the potential to suppress chronic ulcerative colitis-associated colorectal adenocarcinoma development, as shown by inhibition of cellular damage and inflammation-related hyperproliferation [66]. The evidence strongly suggests an NAC benefit in colitis, with alleviation of colitis-induced inflammation, oxidative stress, and tissue damage. The effects of NAC have been reported to be correlated with PI3K/Akt/mTOR, epithelial growth factor receptor, and TLR4/NF-κB signaling, AMP-activated protein kinase (AMPK), and type I interferon [102,105].

### 3.5. Threonine

Threonine is an essential amino acid necessary for intestinal mucosal protein synthesis, especially mucin, and for intestinal integrity, immune barrier function, and oxidative status [106,107]. Supplementation with 1 or 3 g/kg threonine for 21 days resulted in an increase in intestinal villus height, goblet cell density, mucin-2 expression, and immunoglobulin G, immunoglobulin M and secretory immunoglobulin A concentrations in young broiler chickens [107]. Threonine also decreased *Escherichia coli* and *Salmonella* colonies, and increased the *Lactobacillus* colonies in the cecal contents [107]. The mechanisms of threonine-induced protection of intestinal epithelial cells may involve cytoskeletal stabilization, HSP upregulation and nuclear translocation, and a decrease in apoptosis [108]. Pathological conditions (e.g., ileitis and sepsis) may increase the amount of threonine required by the gastrointestinal tract [106]. In a TNBS-induced minipig ileitis model, Rémond et al. [20] reported that intestinal inflammation increased the gastrointestinal tract requirement for threonine. Indeed, generation of mucosal layer and mucin are often quantitatively and qualitatively impaired in IBD [109] and the increased threonine requirement reflects the increased demand for mucin production during intestinal inflammation. Faure et al. [110] reported that increased feeding of an amino acid cocktail containing threonine promoted mucin production and a healthy microbiota and favor colonic protection and mucosal healing in DSS-treated rats. An adequate threonine supply appears crucial to restoration of intestinal integrity during IBD and therefore to enhance recovery.

### 3.6. Tryptophan

Tryptophan regulates intestinal intracellular protein turnover, expression of tight junction proteins [111] and microbiota diversity [112], and reduces intestinal inflammation [113]. It is a precursor of serotonin (5-hydrotryptamine) [67], which is associated with the pathogenesis of experimental colitis [114]. Decreased serum tryptophan observed in IBD patients was shown to reflect a reduction in availability of tryptophan and its metabolites in gut cells and/or an increase in consumption during the inflammatory process [115]. The effect of low serum tryptophan on serotonin biosynthesis can result in impaired quality of life and depression, and tryptophan deficiency may result in immunodeficiency in the presence of persistent immune activation [116]. Tryptophan seems to have a role in IBD, as tryptophan treatment attenuates the symptoms and severity of DSS-induced colitis. In a mouse IBD model, a tryptophan diet ameliorated body weight loss and frequency of bloody stools [67]. It also improved histological evidence of colitis, and decreased the nitrotyrosine content of colon tissues following DSS treatment [67]. A study of its effects on mucosal inflammation found that tryptophan supplementation reduced gut permeability, expression of proinflammatory cytokines, and increased expression of the apoptosis initiators caspase-8 and Bax [68]. In a study comparing the responses of wild-type and aryl hydrocarbon receptor (Ahr)-deficient mice, Islam et al. [69] found that colitis symptoms and production of intestinal inflammatory cytokines were suppressed by activation of Ahr with a 0.5% tryptophan diet. Ahr is a key transcription factor involved in the regulation of inflammation and immunity. Tryptophan was also associated with increased IL-22 and STAT3 expression and protection of epithelial integrity [69]. Thus, tryptophan could be considered as a promising candidate for the treatment of IBD.

### 3.7. Glycine

Glycine, although can be synthesized from serine, is a nutritionally essential amino acid for neonatal growth and development, and appears to protect against ischemic-reperfusion injury [117], oxidative stress [118], endotoxemia [119], and necrotizing enterocolitis [120]. It has been shown to have anti-inflammatory, immunomodulatory, and direct cytoprotective activity [121,122,123]. We previously demonstrated that glycine administration increased intestinal protein content, protein/DNA, RNA/DNA, and villus height/crypt depth ratios and the activities of several tricarboxylic acid cycle enzymes (citrate synthase, isocitrate dehydrogenase and the α-ketoglutarate dehydrogenase complex) under inflammatory conditions [122]. Glycine downregulated proinflammatory cytokines and enhanced protein mass via regulation of TLR4, NOD, AMPK and mTOR signaling [122]. The experimental evidence is sufficient to support a hypothesis that glycine can protect against IBD. Tsune et al. [70] confirmed that glycine alleviated diarrhea, body weight loss, ulceration, and inflammatory changes in the colon caused by TNBS and DSS in animal models. TNBS- or DSS-induced increases in colon MPO activity, proinflammatory IL-1β and TNF-α cytokines and cytokine-induced neutrophil chemoattractant and MIP-2 chemokine secretion were largely blunted in animals fed glycine-supplemented diets [70]. These results suggest that glycine might have prophylactic and therapeutic activity against colitis; data on the effect of glycine on IBD is still limited.

### 3.8. Histidine

Histidine is a conditionally essential amino acid, and is an efficient scavenger of hydroxyl radicals and singlet oxygen [124]. Histidine decarboxylase initiates an immunologically significant pathway for histidine utilization by generating histamine, which can affect acute and chronic inflammation and regulate key events of the immune response [125]. Histidine supplementation can suppress oxidative stress- and TNF-α-induced IL-8 secretion in intestinal epithelial cells [126]. Hasegawa et al. [127] reported that histidine exhibited anti-inflammatory effects in THP-1 human monocytic leukemia cells, and speculated that histidine may also have therapeutic efficacy in IBD. Consistent with this, dietary histidine ameliorated colitis in an IL-10-deficient (IL-10^−/−^) cell transfer model of Crohn’s disease, by regulation of NF-κB activation, and subsequent inhibition of proinflammatory cytokine production by macrophages [71]. Other recent results suggest that decreased plasma histidine predicts risk of relapse in patients with ulcerative colitis in remission [128]. The collective evidence suggests that histidine may be an effective agent in treating various immunopathologic conditions.

### 3.9. Other Amino Acids

Aspartate, asparagine and proline also participate in immune responses [129] that may maintain intestinal health and protect against animal and human diseases. We previously demonstrated that aspartate and asparagine improved intestinal tissue integrity, enhanced mucosal energy status via AMPK signaling, and reduced proinflammatory cytokine expression (via TLR4, NODs and p38) and enterocyte apoptosis (via p38 and extracellular signal-regulated kinase 1/2) in pathological conditions [130,131,132,133]. Proline inhibited lipopolysaccharide-induced inflammation in vivo in weaned piglets [134]. Dietary threonine, serine, proline, and cysteine supplementation improved mucin production, restored healthy microbiota, protected gut epithelium and promoted mucosal healing in DSS-treated rats [110].

Experimental studies investigating the efficacy of amino acids such as glutamine and arginine in animal models of IBD are promising. Some clinical studies with oral supplementation of glutamine in IBD patients are disappointing, but new formulations and targeting at the site of mucosal lesions could improve the efficacy of glutamine [135]. The role of arginine in IBD patients still remains controversial [135]. However, the effects of these amino acids in IBD patients have been poorly investigated. The clinical effects of other amino acids should also be investigated in the future.

## 4. Conclusions

IBD is a complex disease with complex pathogenesis, and no single animal model can fully reflect the entire spectrum of human IBD phenotypes. However, the available models are useful for the study of various aspects and mechanisms of IBD pathogenesis. Amino acids may be necessary for maintaining normal immunocompetence and protecting against human and animal diseases. We predict that these nutrients will likely be used as alternative or ancillary IBD treatments. Additional research is also warranted to add to the understanding of the underlying mechanisms of IBD, and to investigate the clinical effects of these amino acids.

## Figures and Tables

**Table 1:** Examples of chemical-induced inflammatory bowel disease (IBD) animal models.

Colitis Models	Procedure	Animals	References
DSS	1.5–5% (wt/vol) DSS (molecular weight 36–50 kDa) in the drinking water for 5–7 days	rodent	[9,10,11,12,13,14,15]
	1.25 g DSS per kg body weight twice a day for 5 days	piglets	[16]
	Chronic colitis: cyclic treatment with 2% DSS repeated 3 times for 30 days	C57BL/6 mice	[9]
TNBS	2 mg/100 mL of TNBS in 45% ethanol	C57BL/6 mice	[9]
	30 mg TNBS in 0.25 mL 50% ethanol	Wistar rats	[17]
	15 mg TNBS in 0.6 mL 50% ethanol	Sprague-Dawley rats	[18]
	15 mg/kg TNBS in 5 mL 50% ethanol	piglets	[19]
	30-mL of 120 mg/kg TNBS in 50% ethanol	minipigs	[20]
	10 mL of 100% ethanol and 1 g of TNBS in 10 mL of distilled water	adult mongrel dogs	[21]
acetic acid	4% acetic acid for 15 s	rats	[22]
	1 mL of 4% acetic acid in 0.9% NaCl	rats	[23,24]

DSS, dextran sulfate sodium; TNBS, trinitrobenzene sulfonic acid.

**Table 2:** Summary of the effect of amino acid supplementation on IBD in animal models.

Amino Acids	Effects	Animal Models	References
Glutamine	↓body weight loss, colon edema, endothelial adhesion molecules, infiltrating Th cells, PSGL-1, LFA-1, CCR9	C57BL/6 mice; DSS	[10]
	↓epithelium injury and loss, mucosal hypoplasia	Sprague-Dawley rats; acetic acid	[60]
	↓disruption of colonic architecture, submucosal and serosal fibrosis, collagen Iα2, collagen III, TGFβ, phosphorylated Smad3, PDGF, CTGF	Wistar rats; TNBS	[17]
	↑HO-1, GSH ↓MDA, caspase-3, NF-κB	Wistar-albino rats; TNBS	[44]
	↑GSH ↓MDA, caspase-3	Wistar-albino rats; TNBS	[61]
	↓histopathological scores, cytosolic concentration of TBARS, hydroperoxide-initiated chemiluminescence, NF-κB, iNOS, COX-2	Wistars rats; acetic acid	[49]
	↓histopathological scores, cytosolic concentration of TBARS, hydroperoxide-initiated chemiluminescence, MPO, iNOS, COX-2, NF-κB, TNF-α, IFN-γ, phosphorylated forms of STAT1, STAT5, Akt	Wistar rats; TNBS	[62]
	↑Th22 and Treg cell expression ↓Th1/Th17-associated cytokine expression	C57BL/6 mice; DSS	[11]
	↓NF-κB, PI3K-Akt	ICR mice; DSS	[12]
	↑HSP25, HSP70 ↓diarrhea	Sprague-Dawley rats; DSS	[15]
	↓oxidative stress, ER stress, apoptosis	Wistar rats; TNBS	[45]
Glutamate	↑PCNA-positive cells, SOD, Bcl-2 ↓MDA, Bax, caspase-3, TNF-α, IL-1β	Sprague-Dawley rats; TNBS	[63]
Arginine	↑iNOS ↓mucosal permeability, number of MPO-positive neutrophils, and expression of pro-inflammatory cytokine and chemokine	iNOS^−/−^ C57BL/6 mice; DSS	[14]
	↑T-SOD ↓Akt, MLCK	ICR mice; DSS	[12]
	↓body weight loss, colon weights, macroscopic and microscopic damage of colonic tissues	Wistar rats; acetic acid	[64]
Sulphur-containing amino acids	↓colon lesions, cytoskeleton damage, serum amyloid A, TNF-α	BALB/C mice; DSS	[27]
	↑caspase-8 ↓chemokine, neutrophil influx, TNF-α, IL-6, IL-12p40, IL-1β, cFLIP and Bcl-xL	Yorkshire piglets; DSS	[16]
	↑goblet cell number, protein/DNA ratio, claudin-1 ↓IEL number, caspase-3, MPO, MDA, TNF-α	piglets; acetic acid	[54]
	↑GSH, SOD, CAT ↓MPO, MDA	Wistar-albino rats; acetic acid	[65]
	↑PON1,GSH ↓macroscopic colonic damage, histopathologic changes, MPO, ROS, TNF-α, IL-1β	BALB/c mice; DSS	[26]
	↑GSH, SOD ↓MPO, MDA, TNF-α, IL-1β, IL-6	Wistar albino rats; acetic acid	[24]
	↓COX2, PGE_2_	Sprague-Dawley rats; TNBS	[18]
	↓chronic ulcerative colitis-associated colorectal adenocarcinoma development	C57BL/6J mice; DSS	[66]
Tryptophan	↓body weight loss, frequency of bloody stools, nitrotyrosine content of the colonic tissues	C57black6 mice; DSS	[67]
	↑caspase-8, Bax ↓gut permeability, TNF-α, IL-6, IFN-γ, IL-12p40, IL-1β, IL-17, IL-8, ICAM-1	Piglets; DSS	[68]
	↑Ahr, IL-22, STAT3 ↓colitis symptoms, IL-6, TNF-α, IL-1β, Ccl2, Cxcl1, Cxcl2	C57BL/6 WT and KO mice; DSS	[69]
Glycine	↓diarrhea, body weight loss, ulceration, MPO, IL-1β, TNF-α, CINC, MIP-2	Wistar rats; TNBS/DSS	[70]
Histidine	↓histologic damage, colon weight, TNF-α, IL-6, NF-κB	IL-10^−/−^ mice	[71]

Ahr, aryl hydrocarbon receptor; Akt, protein kinase B; Bcl, B-cell lymphoma; Bax, Bcl-2-associated X protein; CAT, catalase; CCR9, C-C chemokine receptor type 9; cFLIP, FLICE-like inhibitory protein; CINC, cytokine-induced neutrophil chemoattractant; COX-2, cyclooxygenase-2; CTGF, connective tissue growth factor; DSS, dextran sulfate sodium; ER, endoplasmic reticulum; GSH, glutathione; HO-1, heme oxygenase-1; HSP, heat shock protein; ICAM-1, intracellular adhesion molecule-1; ICR, institute for cancer research; IEL, intraepithelial lymphocyte; IFN, interferon; IL, interleukin; iNOS, inducible nitric oxide synthase; LFA, leukocyte function-associated antigen; MDA, malondialdehyde; MIP-2, macrophage inflammatory protein-2; MLCK, myosin light chain kinase; MPO, myeloperoxidase; NF-κB, nuclear factor-κB; PCNA, proliferating cell nuclear antigen; PDGF, platelet-derived growth factor; PGE_2_, prostaglandin E_2_; PI3K, phosphoinositide-3-kinases; PON1, paraoxonase 1; PSGL, P-selectin glycoprotein ligand; ROS, reactive oxygen species; SOD, superoxide dismutase; STAT, signal transducers and activators of transcription; TBARS, thiobarbituric acid reactive substances; TGF-β, transforming growth factor-β; Th, T-helper; TNBS, trinitrobenzene sulfonic acid; TNF-α, tumor necrosis factor-α; Treg, T regulatory.

## References

[1] Shi X.Z., Winston J.H., Sarna S.K. (2011). Differential immune and genetic responses in rat models of Crohn’s colitis and ulcerative colitis. Am. J. Physiol. Gastrointest. Liver Physiol..

[2] Randhawa P.K., Singh K., Singh N., Jaggi A.S. (2014). A review on chemical-induced inflammatory bowel disease models in rodents. Korean J. Physiol. Pharmacol..

[3] Snider A.J., Bialkowska A.B., Ghaleb A.M., Yang V.W., Obeid L.M., Hannun Y.A. (2016). Murine model for colitis-associated cancer of the colon. Methods Mol. Biol..

[4] Corridoni D., Arseneau K.O., Cominelli F. (2014). Inflammatory bowel disease. Immunol. Lett..

[5] Goyal N., Rana A., Ahlawat A., Bijjem K.R., Kumar P. (2014). Animal models of inflammatory bowel disease: A review. Inflammopharmacology.

[6] Wu G. (1998). Intestinal mucosal amino acid catabolism. J. Nutr..

[7] Vidal-Lletjós S., Beaumont M., Tomé D., Benamouzig R., Blachier F., Lan A. (2017). Dietary protein and amino acid supplementation in inflammatory bowel disease course: What impact on the colonic mucosa?. Nutrients.

[8] Dai Z., Wu Z., Hang S., Zhu W., Wu G. (2015). Amino acid metabolism in intestinal bacteria and its potential implications for mammalian reproduction. Mol. Hum. Reprod..

[9] Oh S.Y., Cho K.A., Kang J.L., Kim K.H., Woo S.Y. (2014). Comparison of experimental mouse models of inflammatory bowel disease. Int. J. Mol. Med..

[10] Hou Y.C., Wu J.M., Wang M.Y., Wu M.H., Chen K.Y., Yeh S.L., Lin M.T. (2014). Glutamine supplementation attenuates expressions of adhesion molecules and chemokine receptors on T cells in a murine model of acute colitis. Mediat. Inflamm..

[11] Hsiung Y.C., Liu J.J., Hou Y.C., Yeh C.L., Yeh S.L. (2014). Effects of dietary glutamine on the homeostasis of CD4^+^ T cells in mice with dextran sulfate sodium-induced acute colitis. PLoS ONE.

[12] Ren W., Yin J., Wu M., Liu G., Yang G., Xion Y., Su D., Wu L., Li T., Chen S. (2014). Serum amino acids profile and the beneficial effects of l-arginine or l-glutamine supplementation in dextran sulfate sodium colitis. PLoS ONE.

[13] Chu C.C., Hou Y.C., Pai M.H., Chao C.J., Yeh S.L. (2012). Pretreatment with alanyl-glutamine suppresses T-helper-cell-associated cytokine expression and reduces inflammatory responses in mice with acute DSS-induced colitis. J. Nutr. Biochem..

[14] Coburn L.A., Gong X., Singh K., Asim M., Scull B.P., Allaman M.M., Williams C.S., Rosen M.J., Washington M.K., Barry D.P. (2012). l-arginine supplementation improves responses to injury and inflammation in dextran sulfate sodium colitis. PLoS ONE.

[15] Xue H., Sufit A.J., Wischmeyer P.E. (2011). Glutamine therapy improves outcome of in vitro and in vivo experimental colitis models. JPEN J. Parenter. Enteral Nutr..

[16] Kim C.J., Kovacs-Nolan J., Yang C., Archbold T., Fan M.Z., Mine Y. (2009). l-cysteine supplementation attenuates local inflammation and restores gut homeostasis in a porcine model of colitis. Biochim. Biophys. Acta.

[17] San-Miguel B., Crespo I., Kretzmann N.A., Mauriz J.L., Marroni N., Tuñón M.J., González-Gallego J. (2010). Glutamine prevents fibrosis development in rats with colitis induced by 2,4,6-trinitrobenzene sulfonic acid. J. Nutr..

[18] Ancha H.R., Kurella R.R., McKimmey C.C., Lightfoot S., Harty R.F. (2009). Effects of *N*-acetylcysteine plus mesalamine on prostaglandin synthesis and nitric oxide generation in TNBS-induced colitis in rats. Dig. Dis. Sci..

[19] Pouillart P.R., Dépeint F., Abdelnour A., Deremaux L., Vincent O., Mazière J.C., Madec J.Y., Chatelain D., Younes H., Wils D. (2010). Nutriose, a prebiotic low-digestible carbohydrate, stimulates gut mucosal immunity and prevents TNBS-induced colitis in piglets. Inflamm. Bowel. Dis..

[20] Rémond D., Buffière C., Godin J.P., Mirand P.P., Obled C., Papet I., Dardevet D., Williamson G., Breuillé D., Faure M. (2009). Intestinal inflammation increases gastrointestinal threonine uptake and mucin synthesis in enterally fed minipigs. J. Nutr..

[21] Shibata Y., Taruishi M., Ashida T. (1993). Experimental ileitis in dogs and colitis in rats with trinitrobenzene sulfonic acid—Colonoscopic and histopathologic studies. Gastroenterol. Jpn..

[22] Fabia R., Willén R., Ar’Rajab A., Andersson R., Ahrén B., Bengmark S. (1992). Acetic acid-induced colitis in the rat: A reproducible experimental model for acute ulcerative colitis. Eur. Surg. Res..

[23] Tahan G., Aytac E., Aytekin H., Gunduz F., Dogusoy G., Aydin S., Tahan V., Uzun H. (2011). Vitamin E has a dual effect of anti-inflammatory and antioxidant activities in acetic acid-induced ulcerative colitis in rats. Can. J. Surg..

[24] Uraz S., Tahan G., Aytekin H., Tahan V. (2013). *N*-acetylcysteine expresses powerful anti-inflammatory and antioxidant activities resulting in complete improvement of acetic acid-induced colitis in rats. Scand. J. Clin. Lab. Investig..

[25] Kitajima S., Takuma S., Morimoto M. (2000). Histological analysis of murine colitis induced by dextran sulfate sodium of different molecular weights. Exp. Anim..

[26] You Y., Fu J.J., Meng J., Huang G.D., Liu Y.H. (2009). Effect of *N*-acetylcysteine on the murine model of colitis induced by dextran sodium sulfate through up-regulating PON1 activity. Dig. Dis. Sci..

[27] Oz H.S., Chen T.S., McClain C.J., de Villiers W.J. (2005). Antioxidants as novel therapy in a murine model of colitis. J. Nutr. Biochem..

[28] Håkansson Å., Tormo-Badia N., Baridi A., Xu J., Molin G., Hagslätt M.-L., Karlsson C., Jeppsson B., Cilio C.M., Ahrné S. (2015). Immunological alteration and changes of gut microbiota after dextran sulfate sodium (DSS) administration in mice. Clin. Exp. Med..

[29] Munyaka P.M., Rabbi M.F., Khafipour E., Ghia J.-E. (2016). Acute dextran sulfate sodium (DSS)-induced colitis promotes gut microbial dysbiosis in mice. J. Basic Microbiol..

[30] Perše M., Cerar A. (2012). Dextran sodium sulphate colitis mouse model: Traps and tricks. J. Biomed. Biotechnol..

[31] Kiesler P., Fuss I.J., Strober W. (2015). Experimental models of inflammatory bowel diseases. Cell Mol. Gastroenterol. Hepatol..

[32] Vicario M., Amat C., Rivero M., Moretó M., Pelegrí C. (2007). Dietary glutamine affects mucosal functions in rats with mild DSS-induced colitis. J. Nutr..

[33] Amrouche-Mekkioui I., Djerdjouri B. (2012). *N*-acetylcysteine improves redox status, mitochondrial dysfunction, mucin-depleted crypts and epithelial hyperplasia in dextran sulfate sodium-induced oxidative colitis in mice. Eur. J. Pharmacol..

[34] Dagenais M., Douglas T., Saleh M. (2014). Role of programmed necrosis and cell death in intestinal inflammation. Curr. Opin. Gastroenterol..

[35] Qu C., Yuan Z.W., Yu X.T., Huang Y.F., Yang G.H., Chen J.N., Lai X.P., Su Z.R., Zeng H.F., Xie Y. (2017). Patchouli alcohol ameliorates dextran sodium sulfate-induced experimental colitis and suppresses tryptophan catabolism. Pharmacol. Res..

[36] Günther C., Martini E., Wittkopf N., Amann K., Weigmann B., Neumann H., Waldner M.J., Hedrick S.M., Tenzer S., Neurath M.F. (2011). Caspase-8 regulates TNF-α-induced epithelial necroptosis and terminal ileitis. Nature.

[37] Liu Z.Y., Wu B., Guo Y.S., Zhou Y.H., Fu Z.G., Xu B.Q., Li J.H., Jing L., Jiang J.L., Tang J., Chen Z.N. (2015). Necrostatin-1 reduces intestinal inflammation and colitis-associated tumorigenesis in mice. Am. J. Cancer Res..

[38] Welz P.S., Wullaert A., Vlantis K., Kondylis V., Fernández-Majada V., Ermolaeva M., Kirsch P., Sterner-Kock A., van Loo G., Pasparakis M. (2011). FADD prevents RIP3-mediated epithelial cell necrosis and chronic intestinal inflammation. Nature.

[39] Motavallian-Naeini A., Andalib S., Rabbani M., Mahzouni P., Afsharipour M., Minaiyan M. (2012). Validation and optimization of experimental colitis induction in rats using 2,4,6-trinitrobenzene sulfonic acid. Res. Pharm. Sci..

[40] Zheng L., Gao Z.Q., Wang S.X. (2000). A chronic ulcerative colitis model in rats. World J. Gastroenterol..

[41] Tran C.D., Katsikeros R., Abimosleh S.M. (2012). Current and Novel Treatments for Ulcerative Colitis. Ulcerative Colitis from Genetics to Complications.

[42] Antoniou E., Margonis G.A., Angelou A., Pikouli A., Argiri P., Karavokyros I., Papalois A., Pikoulis E. (2016). The TNBS-induced colitis animal model: An overview. Ann. Med. Surg..

[43] Wang J., Chen H., Wang Y., Cai X., Zou M., Xu T., Wang M., Wang J., Xu D. (2016). Therapeutic efficacy of a mutant of keratinocyte growth factor-2 on trinitrobenzene sulfonic acid-induced rat model of Crohn’s disease. Am. J. Transl. Res..

[44] Giriş M., Erbil Y., Doğru-Abbasoğlu S., Yanik B.T., Aliş H., Olgaç V., Toker G.A. (2007). The effect of heme oxygenase-1 induction by glutamine on TNBS-induced colitis. The effect of glutamine on TNBS colitis. Int. J. Colorectal. Dis..

[45] Crespo I., San-Miguel B., Prause C., Marroni N., Cuevas M.J., González-Gallego J., Tuñón M.J. (2012). Glutamine treatment attenuates endoplasmic reticulum stress and apoptosis in TNBS-induced colitis. PLoS ONE.

[46] Siddiqui A., Ancha H., Tedesco D., Lightfoot S., Stewart C.A., Harty R.F. (2006). Antioxidant therapy with *N*-acetylcysteine plus mesalamine accelerates mucosal healing in a rodent model of colitis. Dig. Dis. Sci..

[47] Low D., Nguyen D.D., Mizoguchi E. (2013). Animal models of ulcerative colitis and their application in drug research. Drug Des. Devel. Ther..

[48] Nakao K., Ro A., Kibayashi K. (2014). Evaluation of the morphological changes of gastric mucosa induced by a low concentration of acetic acid using a rat model. J. Forensic Leg. Med..

[49] Fillmann H., Kretzmann N.A., San-Miguel B., Llesuy S., Marroni N., González-Gallego J., Tuñón M.J. (2007). Glutamine inhibits over-expression of pro-inflammatory genes and down-regulates the nuclear factor kappaB pathway in an experimental model of colitis in the rat. Toxicology.

[50] Sakthivel K.M., Guruvayoorappan C. (2013). Amentoflavone inhibits iNOS, COX-2 expression and modulates cytokine profile, NF-κB signal transduction pathways in rats with ulcerative colitis. Int. Immunopharmacol..

[51] Aleisa A.M., Al-Rejaie S.S., Abuohashish H.M., Ola M.S., Parmar M.Y., Ahmed M.M. (2014). Pretreatment of Gymnema sylvestre revealed the protection against acetic acid-induced ulcerative colitis in rats. BMC Complement. Altern. Med..

[52] Niu X., Fan T., Li W., Huang H., Zhang Y., Xing W. (2013). Protective effect of sanguinarine against acetic acid-induced ulcerative colitis in mice. Toxicol. Appl. Pharmacol..

[53] Harisa G.E., Abo-Salem O.M., El-Sayed S.M., Taha E.I., El-Halawany N. (2009). l-arginine augments the antioxidant effect of garlic against acetic acid-induced ulcerative colitis in rats. Pak. J. Pharm. Sci..

[54] Wang Q., Hou Y., Yi D., Wang L., Ding B., Chen X., Long M., Liu Y., Wu G. (2013). Protective effects of *N*-acetylcysteine on acetic acid-induced colitis in a porcine model. BMC Gastroenterol..

[55] Ali A.A., Abd Al Haleem E.N., Khaleel S.A., Sallam A.S. (2017). Protective effect of cardamonin against acetic acid-induced ulcerative colitis in rats. Pharmacol. Rep..

[56] Wirtz S., Popp V., Kindermann M., Gerlach K., Weigmann B., Fichtner-Feigl S., Neurath M.F. (2017). Chemically induced mouse models of acute and chronic intestinal inflammation. Nat. Protoc..

[57] Berry D., Kuzyk O., Rauch I., Heider S., Schwab C., Hainzl E., Decker T., Müller M., Strobl B., Schleper C. (2015). Intestinal microbiota signatures associated with inflammation history in mice experiencing recurring colitis. Front. Microbiol..

[58] Mowat C., Cole A., Windsor A., Ahmad T., Arnott I., Driscoll R., Mitton S., Orchard T., Rutter M., Younge L. (2011). Guidelines for the management of inflammatory bowel disease in adults. Gut.

[59] Xin L., Li N., Zhu W., Wu K., Zhang L., Zhai J., Wang Y., Zhu J., Wang X., Shi Y. (2015). An analysis of amino acid metabolic profile and its clinical significance in ulcerative colitis. Zhonghua Nei Ke Za Zhi.

[60] Swaid F., Sukhotnik I., Matter I., Berkowitz D., Hadjittofi C., Pollak Y., Lavy A. (2013). Dietary glutamine supplementation prevents mucosal injury and modulates intestinal epithelial restitution following acetic acid induced intestinal injury in rats. Nutr. Metab..

[61] Değer C., Erbil Y., Giriş M., Yanik B.T., Tunca F., Olgaç V., Abbasoğlu S.D., Oztezcan S., Toker G. (2006). The effect of glutamine on pancreatic damage in TNBS-induced colitis. Dig. Dis. Sci..

[62] Kretzmann N.A., Fillmann H., Mauriz J.L., Marroni C.A., Marroni N., González-Gallego J., Tuñón M.J. (2008). Effects of glutamine on proinflammatory gene expression and activation of nuclear factor kappa B and signal transducers and activators of transcription in TNBS-induced colitis. Inflamm. Bowel Dis..

[63] Li T.T., Zhang J.F., Fei S.J., Zhu S.P., Zhu J.Z., Qiao X., Liu Z.B. (2014). Glutamate microinjection into the hypothalamic paraventricular nucleus attenuates ulcerative colitis in rats. Acta Pharmacol. Sin..

[64] Farghaly H.S., Thabit R.H. (2014). l-arginine and aminoguanidine reduce colonic damage of acetic acid-induced colitis in rats: Potential modulation of nuclear factor-κB/p65. Clin. Exp. Pharmacol. Physiol..

[65] Cetinkaya A., Bulbuloglu E., Kurutas E.B., Ciralik H., Kantarceken B., Buyukbese M.A. (2005). Beneficial effects of *N*-acetylcysteine on acetic acid-induced colitis in rats. Tohoku J. Exp. Med..

[66] Seril D.N., Liao J., Ho K.L., Yang C.S., Yang G.Y. (2002). Inhibition of chronic ulcerative colitis-associated colorectal adenocarcinoma development in a murine model by *N*-acetylcysteine. Carcinogenesis.

[67] Shizuma T., Mori H., Fukuyama N. (2013). Protective effect of tryptophan against dextran sulfate sodium- induced experimental colitis. Turk. J. Gastroenterol..

[68] Kim C.J., Kovacs-Nolan J.A., Yang C., Archbold T., Fan M.Z., Mine Y. (2010). l-Tryptophan exhibits therapeutic function in a porcine model of dextran sodium sulfate (DSS)-induced colitis. J. Nutr. Biochem..

[69] Islam J., Sato S., Watanabe K., Watanabe T., Ardiansyah, Hirahara K., Aoyama Y., Tomita S., Aso H., Komai M. (2017). Dietary tryptophan alleviates dextran sodium sulfate-induced colitis through aryl hydrocarbon receptor in mice. J. Nutr. Biochem..

[70] Tsune I., Ikejima K., Hirose M., Yoshikawa M., Enomoto N., Takei Y., Sato N. (2003). Dietary glycine prevents chemical-induced experimental colitis in the rat. Gastroenterology.

[71] Andou A., Hisamatsu T., Okamoto S., Chinen H., Kamada N., Kobayashi T., Hashimoto M., Okutsu T., Shimbo K., Takeda T. (2009). Dietary histidine ameliorates murine colitis by inhibition of proinflammatory cytokine production from macrophages. Gastroenterology.

[72] Sido B., Seel C., Hochlehnert A., Breitkreutz R., Dröge W. (2006). Low intestinal glutamine level and low glutaminase activity in Crohn’s disease: A rational for glutamine supplementation?. Dig. Dis. Sci..

[73] Fasina Y.O., Bowers J.B., Hess J.B., McKee S.R. (2010). Effect of dietary glutamine supplementation on Salmonella colonization in the ceca of young broiler chicks. Poult. Sci..

[74] Zhang Y., Lu T., Han L., Zhao L., Niu Y., Chen H. (2017). l-glutamine supplementation alleviates constipation during late gestation of mini sows by modifying the microbiota composition in feces. BioMed Res. Int..

[75] Benjamin J., Makharia G., Ahuja V., Anand Rajan K.D., Kalaivani M., Gupta S.D., Joshi Y.K. (2012). Glutamine and whey protein improve intestinal permeability and morphology in patients with Crohn’s disease: A randomized controlled trial. Dig. Dis. Sci..

[76] Ockenga J., Borchert K., Stüber E., Lochs H., Manns M.P., Bischoff S.C. (2005). Glutamine-enriched total parenteral nutrition in patients with inflammatory bowel disease. Eur. J. Clin. Nutr..

[77] Den Hond E., Hiele M., Peeters M., Ghoos Y., Rutgeerts P. (1999). Effect of long-term oral glutamine supplements on small intestinal permeability in patients with Crohn’s disease. JPEN J. Parenter. Enter. Nutr..

[78] Akobeng A.K., Elawad M., Gordon M. (2016). Glutamine for induction of remission in Crohn’s disease. Cochrane Database Syst. Rev..

[79] Newsholme P., Lima M.M., Procopio J., Pithon-Curi T.C., Doi S.Q., Bazotte R.B., Curi R. (2003). Glutamine and glutamate as vital metabolites. Braz. J. Med. Biol. Res..

[80] Tapiero H., Mathé G., Couvreur P., Tew K.D. (2002). Glutamine and glutamate. Biomed. Pharmacother..

[81] Blachier F., Boutry C., Bos C., Tomé D. (2009). Metabolism and functions of l-glutamate in the epithelial cells of the small and large intestines. Am. J. Clin. Nutr..

[82] Jiao N., Wu Z., Ji Y., Wang B., Dai Z., Wu G. (2015). l-Glutamate enhances barrier and antioxidative functions in intestinal porcine epithelial cells. J. Nutr..

[83] Wu M., Xiao H., Ren W., Yin J., Tan B., Liu G., Li L., Nyachoti C.M., Xiong X., Wu G. (2014). Therapeutic effects of glutamic acid in piglets challenged with deoxynivalenol. PLoS ONE.

[84] Wang X.Y. (2015). Regulative Effect of Glutamate on Intestinal Injury and Muscle Protein Synthesis and Degradation of Piglets after Lipopolysaccharide Challenge. Master’s Thesis.

[85] Ren X.R. (2015). Regulatory Effect of Glutamic Acid or Glycine on Intestinal Mucosal Immune Barrier Injury in Piglets after Lipopolysaccharide Challenge. Master’s Thesis.

[86] Ren W., Chen S., Yin J., Duan J., Li T., Liu G., Feng Z., Tan B., Yin Y., Wu G. (2014). Dietary arginine supplementation of mice alters the microbial population and activates intestinal innate immunity. J. Nutr..

[87] Wu G., Meininger C.J., Knabe D.A., Bazer F.W., Rhoads J.M. (2000). Arginine nutrition in development, health and disease. Curr. Opin. Clin. Nutr. Metab. Care.

[88] Coburn L.A., Horst S.N., Allaman M.M., Brown C.T., Williams C.S., Hodges M.E., Druce J.P., Beaulieu D.B., Schwartz D.A., Wilson K.T. (2016). l-Arginine availability and metabolism is altered in ulcerative colitis. Inflamm. Bowel Dis..

[89] Zhu H.L., Liu Y.L., Xie X.L., Huang J.J., Hou Y.Q. (2013). Effect of l-arginine on intestinal mucosal immune barrier function in weaned pigs after *Escherichia coli* LPS challenge. Innate Immun..

[90] Liu Y.L., Han J., Huang J.J., Wang X.Q., Wang F.L., Wang J.J. (2009). Dietary l-Arginine supplementation improves intestinal function in weaned pigs after an *Escherichia coli* lipopolysaccharide. Asian-Aust. J. Anim. Sci..

[91] Liu Y., Huang J., Hou Y., Zhu H., Zhao S., Ding B., Yin Y., Yi G., Shi J., Fan W. (2008). Dietary arginine supplementation alleviates intestinal mucosal disruption induced by *Escherichia coli* lipopolysaccharide in weaned pigs. Br. J. Nutr..

[92] Lecleire S., Hassan A., Marion-Letellier R., Antonietti M., Savoye G., Bôle-Feysot C., Lerebours E., Ducrotté P., Déchelotte P., Coëffier M. (2008). Combined glutamine and arginine decrease proinflammatory cytokine production by biopsies from Crohn’s patients in association with changes in nuclear factor-kappaB and p38 mitogen-activated protein kinase pathways. J. Nutr..

[93] Chen Y., Li D., Dai Z., Piao X., Wu Z., Wang B., Zhu Y., Zeng Z. (2014). l-methionine supplementation maintains the integrity and barrier function of the small-intestinal mucosa in post-weaning piglets. Amino Acids.

[94] Shen Y.B., Weaver A.C., Kim S.W. (2014). Effect of feed grade l-methionine on growth performance and gut health in nursery pigs compared with conventional dl-methionine. J. Anim. Sci..

[95] Ramalingam A., Wang X., Gabello M., Valenzano M.C., Soler A.P., Ko A., Morin P.J., Mullin J.M. (2010). Dietary methionine restriction improves colon tight junction barrier function and alters claudin expression pattern. Am. J. Physiol. Cell Physiol..

[96] Komninou D., Leutzinger Y., Reddy B.S., Richie J.P. (2006). Methionine restriction inhibits colon carcinogenesis. Nutr. Cancer.

[97] Rao Y.X., Chen J., Chen L.L., Gu W.Z. (2013). The impact of dietary methionine-restriction on tight junction expression and function in a rat colonitis model. Zhonghua Nei Ke Za Zhi.

[98] Tang Y., Tan B., Xiong X., Li F., Ren W., Kong X., Qiu W., Hardwidge P.R., Yin Y. (2015). Methionine deficiency reduces autophagy and accelerates death in intestinal epithelial cells infected with enterotoxigenic *Escherichia coli*. Amino Acids.

[99] Li T.W., Yang H., Peng H., Xia M., Mato J.M., Lu S.C. (2012). Effects of *S*-adenosylmethionine and methylthioadenosine on inflammation-induced colon cancer in mice. Carcinogenesis.

[100] Ji Y., Wu Z., Dai Z., Sun K., Zhang Q., Wu G. (2016). Excessive l-cysteine induces vacuole-like cell death by activating endoplasmic reticulum stress and mitogen-activated protein kinase signaling in intestinal porcine epithelial cells. Amino Acids.

[101] Song Z.H., Tong G., Xiao K., Jiao L.F., Ke Y.L., Hu C.H. (2016). l-cysteine protects intestinal integrity, attenuates intestinal inflammation and oxidant stress, and modulates NF-κB and Nrf2 pathways in weaned piglets after LPS challenge. Innate Immun..

[102] Hou Y., Wang L., Yi D., Wu G. (2015). *N*-acetylcysteine and intestinal health: A focus on its mechanism of action. Front. Biosci..

[103] Xu C.C., Yang S.F., Zhu L.H., Cai X., Sheng Y.S., Zhu S.W., Xu J.X. (2014). Regulation of *N*-acetyl cysteine on gut redox status and major microbiota in weaned piglets. J. Anim. Sci..

[104] Xie X., Zhao Y., Ma C.Y., Xu X.M., Zhang Y.Q., Wang C.G., Jin J., Shen X., Gao J.L., Li N. (2015). Dimethyl fumarate induces necroptosis in colon cancer cells through GSH depletion/ROS increase/MAPKs activation pathway. Br. J. Pharmacol..

[105] Yi D., Hou Y.Q., Xiao H., Wang L., Zhang Y., Chen H.B., Wu T., Ding B.Y., Hu C.A., Wu G.Y. *N*-Acetylcysteine improves intestinal function in lipopolysaccharides-challenged piglets through multiple signaling pathways. Amino Acids.

[106] Mao X., Zeng X., Qiao S., Wu G., Li D. (2011). Specific roles of threonine in intestinal mucosal integrity and barrier function. Front. Biosci..

[107] Chen Y.P., Cheng Y.F., Li X.H., Yang W.L., Wen C., Zhuang S., Zhou Y.M. (2017). Effects of threonine supplementation on the growth performance, immunity, oxidative status, intestinal integrity, and barrier function of broilers at the early age. Poult. Sci..

[108] Baird C.H., Niederlechner S., Beck R., Kallweit A.R., Wischmeyer P.E. (2013). l-Threonine induces heat shock protein expression and decreases apoptosis in heat-stressed intestinal epithelial cells. Nutrition.

[109] Corfield A.P., Myerscough N., Longman R., Sylvester P., Arul S., Pignatelli M. (2000). Mucins and mucosal protection in the gastrointestinal tract: New prospects for mucins in the pathology of gastrointestinal disease. Gut.

[110] Faure M., Mettraux C., Moennoz D., Godin J.P., Vuichoud J., Rochat F., Breuillé D., Obled C., Corthésy-Theulaz I. (2006). Specific amino acids increase mucin synthesis and microbiota in dextran sulfate sodium-treated rats. J. Nutr..

[111] Wang H., Ji Y., Wu G., Sun K., Sun Y., Li W., Wang B., He B., Zhang Q., Dai Z., Wu Z. (2015). l-Tryptophan activates mammalian target of rapamycin and enhances expression of tight junction proteins in intestinal porcine epithelial cells. J. Nutr..

[112] Messori S., Trevisi P., Simongiovanni A., Priori D., Bosi P. (2013). Effect of susceptibility to enterotoxigenic *Escherichia coli* F4 and of dietary tryptophan on gut microbiota diversity observed in healthy young pigs. Vet. Microbiol..

[113] Etienne-Mesmin L., Chassaing B., Gewirtz A.T. (2017). Tryptophan: A gut microbiota-derived metabolites regulating inflammation. World J. Gastrointest. Pharmacol. Ther..

[114] Ghia J.E., Li N., Wang H., Collins M., Deng Y., El-Sharkawy R.T., Côté F., Mallet J., Khan W.I. (2009). Serotonin has a key role in pathogenesis of experimental colitis. Gastroenterology.

[115] Nikolaus S., Al-Massad N., Bethge J., Waetzig G.H., Rosenstiel P.C., Junker R., Thieme F., Schreiber S. (2014). Su1079 Tryptophan deficiency in Crohn disease. Gastroenterology.

[116] Schröcksnadel K., Wirleitner B., Winkler C., Fuchs D. (2006). Monitoring tryptophan metabolism in chronic immune activation. Clin. Chim. Acta.

[117] Petrat F., Boengler K., Schulz R., de Groot H. (2012). Glycine, a simple physiological compound protecting by yet puzzling mechanism(s) against ischaemia-reperfusion injury: Current knowledge. Br. J. Pharmacol..

[118] Wang W., Wu Z., Lin G., Hu S., Wang B., Dai Z., Wu G. (2014). Glycine stimulates protein synthesis and inhibits oxidative stress in pig small intestinal epithelial cells. J. Nutr..

[119] Effenberger-Neidnicht K., Jägers J., Verhaegh R., de Groot H. (2014). Glycine selectively reduces intestinal injury during endotoxemia. J. Surg. Res..

[120] Meyer K.F., Martins J.L., de Freitas Filho L.G., Oliva M.L., Patrício F.R., Macedo M., Wang L. (2006). Glycine reduces tissue lipid peroxidation in hypoxia-reoxygenation-induced necrotizing enterocolitis in rats. Acta Cir. Bras..

[121] Zhong Z., Wheeler M.D., Li X., Froh M., Schemmer P., Yin M., Bunzendaul H., Bradford B., Lemasters J.J. (2003). l-Glycine: A novel antiinflammatory, immunomodulatory, and cytoprotective agent. Curr. Opin. Clin. Nutr. Metab. Care.

[122] Wu H.T. (2015). Regulative Effect of Glycine on Intestinal Injury and Muscle Protein Sythesis and Degradation of Piglets after Lipopolysaccharide Challenge. Master’s Thesis.

[123] Liu Y., Wang X., Wu H., Chen S., Zhu H., Zhang J., Hou Y., Hu C.A., Zhang G. (2016). Glycine enhances muscle protein mass associated with maintaining Akt-mTOR-FOXO1 signaling and suppressing TLR4 and NOD2 signaling in piglets challenged with LPS. Am. J. Physiol. Regul. Integr. Comp. Physiol..

[124] Wade A.M., Tucker H.N. (1998). Antioxidant characteristics of l-histidine. J. Nutr. Biochem..

[125] Jutel M., Akdis M., Akdis C.A. (2009). Histamine, histamine receptors and their role in immune pathology. Clin. Exp. Allergy.

[126] Son D.O., Satsu H., Shimizu M. (2005). Histidine inhibits oxidative stress- and TNF-alpha-induced interleukin-8 secretion in intestinal epithelial cells. FEBS Lett..

[127] Hasegawa S., Ichiyama T., Sonaka I., Ohsaki A., Hirano R., Haneda Y., Fukano R., Hara M., Furukawa S. (2011). Amino acids exhibit anti-inflammatory effects in human monocytic leukemia cell line, THP-1 cells. Inflamm. Res..

[128] Hisamatsu T., Ono N., Imaizumi A., Mori M., Suzuki H., Uo M., Hashimoto M., Naganuma M., Matsuoka K., Mizuno S. (2015). Decreased plasma histidine level predicts risk of relapse in patients with ulcerative colitis in remission. PLoS ONE.

[129] Li P., Yin Y.L., Li D., Kim S.W., Wu G. (2007). Amino acids and immune function. Br. J. Nutr..

[130] Pi D., Liu Y., Shi H., Li S., Odle J., Lin X., Zhu H., Chen F., Hou Y., Leng W. (2014). Dietary supplementation of aspartate enhances intestinal integrity and energy status in weanling piglets after lipopolysaccharide challenge. J. Nutr. Biochem..

[131] Wang X., Liu Y., Li S., Pi D., Zhu H., Hou Y., Shi H., Leng W. (2015). Asparagine attenuates intestinal injury, improves energy status and inhibits AMP-activated protein kinase signalling pathways in weaned piglets challenged with *Escherichia coli* lipopolysaccharide. Br. J. Nutr..

[132] Wang H., Liu Y., Shi H., Wang X., Zhu H., Pi D., Leng W., Li S. (2017). Aspartate attenuates intestinal injury and inhibits TLR4 and NODs/NF-κB and p38 signaling in weaned pigs after LPS challenge. Eur. J. Nutr..

[133] Chen S., Liu Y., Wang X., Wang H., Li S., Shi H., Zhu H., Zhang J., Pi D., Hu C.A., Lin X., Odle J. (2016). Asparagine improves intestinal integrity, inhibits TLR4 and NOD signaling, and differently regulates p38 and ERK1/2 signaling in weanling piglets after LPS challenge. Innate Immun..

[134] Kang P., Zhang L., Hou Y., Ding B., Yi D., Wang L., Zhu H., Liu Y., Yin Y., Wu G. (2014). Effects of l-proline on the growth performance, and blood parameters in weaned lipopolysaccharide (LPS)-challenged pigs. Asian-Australas. J. Anim. Sci..

[135] Coëffier M., Marion-Letellier R., Déchelotte P. (2010). Potential for amino acids supplementation during inflammatory bowel diseases. Inflamm. Bowel Dis..

